# A comparative study of quality and safety of Atlantic cod (Gadus morhua) fillets during cold storage, as affected by different thawing methods of pre‐rigor frozen headed and gutted fish

**DOI:** 10.1002/jsfa.8649

**Published:** 2017-10-06

**Authors:** Irja Sunde Roiha, Ásbjörn Jónsson, Christoph Josef Backi, Bjørn Tore Lunestad, Magnea G Karlsdóttir

**Affiliations:** ^1^ National Institute of Nutrition and Seafood Research Nordnes, Bergen Norway; ^2^ Matís Ltd. Icelandic Food and Biotech R&D Vinlandsleid 12, Reykjavík Iceland; ^3^ Department of Chemical Engineering Norwegian University of Science and Technology 7491 Trondheim Norway

**Keywords:** whitefish, cod, thawing, quality, shelf life, safety

## Abstract

**BACKGROUND:**

The catch of marine whitefish is typically seasonal, whereas the land‐based processing industry has a need for all‐year stable supply of raw materials. This challenge can be met by applying fish frozen at sea. When using frozen fish, the methods employed for thawing may influence the safety and quality of the final product. This study aimed to investigate the applicability of novel thawing strategies in order to provide an all‐year supply of high‐quality and safe cod products.

**RESULTS:**

Comparative investigations of quality and safety factors after thawing in water, with and without air circulation, and contact thawing were performed. The parameters included water‐holding capacity, thawing loss, drip loss, cooking yield, sensory evaluation and microbiological analyses (including total volatile bases nitrogen). Water thawing with air circulation provided faster thawing than water thawing without air circulation and contact thawing. For all three methods, the quality of the thawed fish was acceptable and the shelf life of the fillets during chilled storage was between 10 and 14 days post‐filleting.

**CONCLUSION:**

The results show that controlled freezing of cod, followed by appropriate thawing, may provide the processing industry with an all‐year delivery of raw materials, without compromising quality and safety of the final product. © 2017 The Authors. *Journal of The Science of Food and Agriculture* published by John Wiley & Sons Ltd on behalf of Society of Chemical Industry.

## INTRODUCTION

A major challenge in the whitefish industry is the seasonal variation in landings throughout the year. While most of the whitefish of the North East Atlantic area are caught and processed during winter, the market demands an all‐year delivery of high‐quality products. This is especially the case for the large Norwegian fisheries of Atlantic cod (*Gadus morhua*), with a short 4‐month commercial season, from mid‐January to mid‐April. The large volumes of fish caught during a limited period lead to processing capacity challenges in the land‐based industry. This challenge can be met by applying fish frozen at sea and subsequent thawing by optimal procedures.

Fish are amongst the most perishable food products, and even at normal refrigeration storage conditions the shelf life is limited by oxidative, enzymatic and microbiological spoilage.^1^, ^2^, ^3^, ^4^ Freezing is a method of preservation, and important for shelf life extension and maintenance of quality. However, the quality of the product is closely related to the freezing and thawing conditions, and the choice of method for thawing has the potential to affect the quality of the fish, as chemical reactions and muscle degradation may escalate.^5^, ^6^, ^7^, ^8^, ^9^, ^10^, ^11^ There are many potential methods for thawing fish,^12^, ^13^, ^14^, ^15^ and methods applicable at an industrial scale were recently reviewed by Backi.^16^

The main specific spoilage bacteria (SSBs) reported in cold water marine fish are *Pseudomonas* spp., *Shewanella putrefaciens* and *Photobacterium phosphoreum*.^17^, ^18^ *P. phosphoreum* is CO_2_ tolerant and is a spoilage organism of fish stored under modified atmosphere, whereas *Pseudomonas* spp. and *S. putrefaciens* produce H_2_S and are the predominant specific spoilage organisms of fresh marine fish from temperate waters stored under aerobic conditions.^19^

The ability of the fish muscle to retain its natural water and, hence, its juiciness is one of the quality criteria of fresh fish, especially from the perspective of the consumer. Therefore, drip loss during storage and after cooking are important parameters that must be included when evaluating seafood quality.

This study was designed to determine the impact on fillet shelf life by different thawing methods of *pre‐rigor* frozen headed and gutted (H/G) Atlantic cod (*Gadus morhua*) during subsequent cold storage. Moreover, special emphasis was placed on assessing how the proposed thawing methods affect food quality and safety. The thawing methods investigated include one contact thawing and two water thawing methods, one with and one without air circulation. For the water thawing methods, the fish was in direct contact with the thawing medium, whereas for the contact thawing method the fish was in contact with a metal plate heated by the thawing medium. Hence, for the latter method, there was no direct contact between the fish and the thawing medium.

## MATERIALS AND METHODS

### Experimental design

Atlantic cod (*G. morhua*) was caught by a commercial trawling vessel in the Barents Sea, in August 2014. Fish from a single haul were H/G and frozen *pre‐rigor* in blocks at −40 °C using a vertical plate freezer on‐board the vessel. Subsequently, the fish blocks were stored at −25 °C for at least 6 weeks to ensure the fish had passed *rigor mortis* before thawing, and therefore thaw rigor could be avoided.^20^ Nine blocks of frozen fish were divided into three groups, where each block contained 26 kg of H/G cod (all fish under 1000 g). Each of the three groups was thawed by applying a different method: water thawing with and without air circulation, and contact thawing by a converted plate freezer. After thawing, the fish were stored at 0 °C overnight before filleting and packaging. Hence, all fish held the same temperature of approximately 0 °C when filleted. The H/G cod were manually filleted skin on and preweighed before packing into expanded polystyrene boxes (Promens Tempra, Hafnarfjordur, Iceland) with plastic film and two cooling mats placed on top and absorbent pads underneath the fish. The boxes accommodated up to 3 kg fish and all samples were stored at 0–2 °C for up to 14 days post‐packaging, and both ambient temperatures and temperatures inside the boxes were recorded at 10 min intervals.

### Thawing procedures

**Figure 1:**
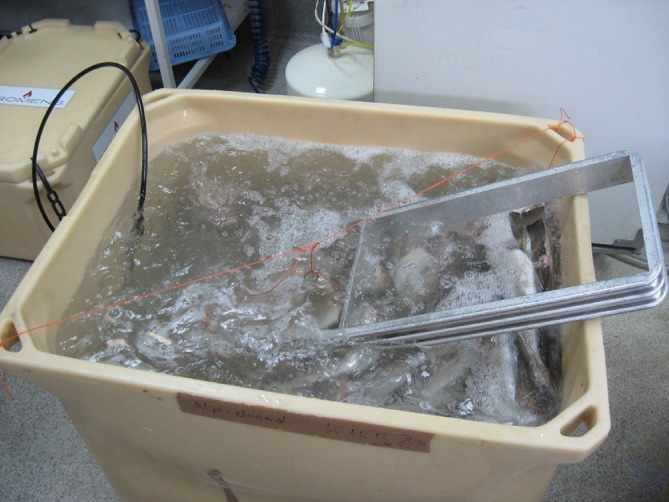
Fish blocks under air‐assisted thawing in a 1000 L fish tub. The air bubbles were generating circulation for enhanced overall heat transfer.

Two experimental groups were thawed by immersion in fresh water using clean 1000 L fish tubs (Promens, Dalvik, Iceland). In one of the tubs, a diffusor element was placed at the bottom, generating air circulation (Fig. 1). The air generated circulation, thus enhancing the overall heat transfer. The initial temperature of the water was set to 18 °C, which ultimately decreased during the thawing process. The water was not replaced during the entire thawing process. The fish‐to‐water ratio was set to 1 : 4, which was calculated according to the following thermodynamic steady‐state equations:

(1) $$q_{\text{fish}}=\left[m_{\text{fish}}c_{p\left(\text{fish}\right)}\left(T_{1}-T_{2}\right)\right]+\left({\mathrm{Lm}}_{\text{fish}}\right)$$

(2) $$q_{\text{water}}=m_{\text{water}}c_{p\left(\text{water}\right)}\left(T_{3}-T_{4}\right)$$

(3) $$q_{\text{fish}}=q_{\text{water}}$$

where m_fish_ is the mass of the fish in the tub, m_water_ is the mass of the water needed, c_p(water)_ is the specific heat of the water (4.18 kJ^−1^ kg^−1^ K^−1^), c_p(fish)_ is the specific heat of the frozen fish (2.1 kJ^−1^ kg^−1^ K^−1^), L is the latent heat of fusion (melting) for the fish (305.4 kJ^−1^ kg^−1^ K^−1^), T_1_ is the initial temperature of the fish (−25 °C), T_2_ is the melting point of lean fish (−1.2 °C), T_3_ is the initial temperature of the water (18 °C) and T_4_ is the final temperature of the water (minimal 0 °C). Note that Eqns (1), (2), (3) represent a situation with uniform distribution of the temperature field inside the spatial domain of the fish. This means that neither spatially nor time‐dependent phenomena can be calculated; compare Backi,^21^ Backi et al.,^22^ or alike.

**Figure 2:**
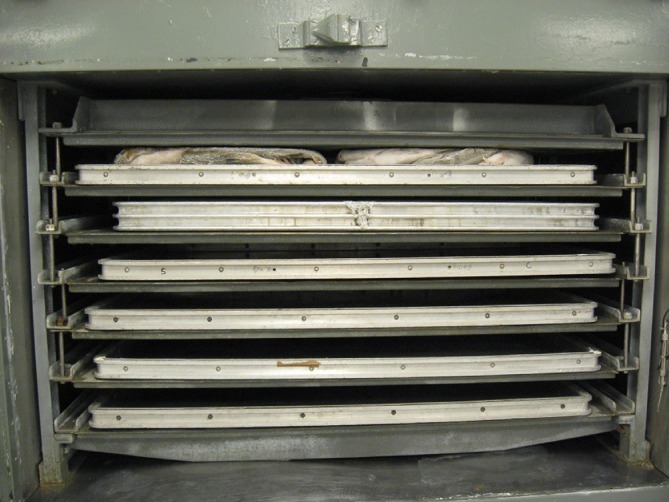
Two fish blocks in a contact thawer, a converted horizontal plate freezer.

For the third experimental group, frozen fish blocks were thawed by a converted horizontal plate freezer (Jackstone Freezing Systems Ltd, Norfolk, UK) (Fig. 2). The converted plate freezer (denoted ‘plate thawer’) was operated with water as thawing medium, which was pumped through the plate freezer walls. The set‐point for the water temperature was defined as 10 °C.

### Temperature profiling

To follow the temperature profile of the fish muscle during thawing, temperature data loggers (iButtons^®^, Maxim Integrated, California, USA) were placed in the frozen fish by drilling a hole of predefined length into the fish block, using a hole‐saw with a diameter of 22 mm (WILPU^®^, Wilh. Putsch GmbH & Co. KG, Germany). Three loggers per group were used, located from right below the surface (<1 cm) of the block to close to the core of the block. The temperature of the thawing water was recorded at approximately 35 cm depth in the middle of the tub and at one of the corners. For temperature profiling of the converted plate freezer, loggers were attached to the heating plates to record the temperature of the heat supply. Temperature recordings were sampled at 5 min intervals.

### Sampling

Sampling and analysis of the fillets were performed after 0, 6, 10 and 14 days' storage post‐packaging. A summary of the analytical programme performed can be viewed in Table 1. All physicochemical and microbiological analyses were set up in triplicate (*n* = 3). For evaluation of cooking loss, a portion of about 50 g was removed from the middle of the fillets, while portions from the loin, middle and tail sections were collected for evaluation of proximate composition, water‐holding capacity (WHC) and microbiological quality. Any deviation from this protocol is included in the methods descriptions.

**Table 1:** Summary of the analysis programme performed on the thawed H/G cod, fillets and thawing media

	Quality parameter	H/G fish	Fillets	Thawing media Before and after thawing
Physicochemical parameters	Temperature	×	×	×
	Thaw drip loss	×		
	Drip loss during chilled storage		×	
	Fillet evaluation		×	
	WHC		×	
	Cooking loss		×	
	Proximate composition		×	
	TVB‐N		×	×
Microbiological parameters	TVC‐IA		×	×
	H_2_S‐IA		×	×
	Total coliforms		×	×
	Thermo‐tolerant coliforms		×	×
	*Listeria monocytogenes*		×	×

### Water‐holding capacity

The WHC was determined by a centrifugation method.^23^ The cod muscle was coarsely minced in a Braun mixer (Type 4262, Germany) for 10–15 s, depending on the size and thickness of the fillets, and 2 g of the sample was weighed in a glass sample holder. Samples were centrifuged at 210 × *g* for 5 min at 4 °C (Heareus Biofuge Stratos Reconditioned 75005289R, Rotor 3335. DJB Labcare Ltd, UK). Centrifugation loss of water was calculated as the difference in weight before and after centrifugation. The WHC (expressed as a percentage) was calculated as the ratio of remaining water compared with the water content in the sample before centrifugation.

### Thawing and drip loss

The drip loss of the thawed fish blocks was determined from the known weights of the fish blocks before and after thawing and expressed as

$$\text{Thawing}\;\text{loss}\;\left(\%\right)=\frac{\text{Weight}\;\text{of}\;\text{frozen}\;\text{fish}\;\text{block}\;\left(g\right)-\text{Weight}\;\text{of}\;\text{thawed}\;\text{fish}\;\text{block}\;\left(g\right)}{\text{Weight}\;\text{of}\;\text{frozen}\;\text{fish}\;\text{block}\;\left(g\right)}\times 100$$

After filleting, the fillets were weighed before packaging, and again after each sampling, to evaluate drip loss during cold storage according to the following equation:

$$\text{Drip}\;\text{loss}\;\left(\%\right)=\frac{\text{Weight}\;\text{of}\;\text{fillets}\;\text{before}\;\text{storage}\;\left(g\right)-\text{Weight}\;\text{of}\;\text{fillets}\;\text{after}\;\text{storage}\;\left(g\right)}{\text{Weight}\;\text{of}\;\text{fillets}\;\text{before}\;\text{storage}\;\left(g\right)}\times 100$$

### Cooking yield

The cooking yield of the fillets was determined directly after filleting and during the storage period. Approximately 50 g of the middle parts of sampled fillets (*n* = 6) with skin were placed on racks and cooked for 8 min in a preheated oven (Convotherm Elektrogeräte GmbH, Eglfing, Germany), at 95 °C with air circulation and steam. After cooking, excess water was separated from the material and the cooked samples were cooled to room temperature (20 °C) for 15 min before additional weighing. Percentage cooking yield was determined by the following equation:

$$\text{Cooking}\;\text{yield}\;\left(\%\right)=\frac{\text{Weight}\;\text{of}\;\text{cooked}\;\text{muscle}\;\left(g\right)}{\text{Weight}\;\text{of}\;\text{uncooked}\;\text{muscle}\;\left(g\right)}\times 100$$

### Sensory evaluation

The quality index method scheme for cod, based on the work of Bonilla *et al*.,^24^ was applied to evaluate the cod fillets directly after filleting and following cold storage. Attributes were adjusted to meet the scope of the current study, and scores were based on colour, texture, gaping and smell. Total score shows the quality index and gives an indication of storage time and remaining shelf life. Every sampling day, six fillets from each experimental group were placed on a white board and evaluated according to the scheme described in Table 2. Five panellists participated in the evaluation. They had all been trained according to international standards^25^ and were experienced in the sensory method used in this evaluation.

**Table 2:** The quality index method scheme used to evaluate the cod fillets for sensory evaluation, adopted from Bonilla et al. 24 with modifications

Quality attribute	Description	Grade
Texture	Firm, springy	0
	Firmness gained slowly after pressure	1
	Soft texture, no springiness	2
Colour	Shining, bright colour according to species	0
	Matte colour, characteristic for species	1
	Small yellow dots, colour very matte/dull	2
	Large yellow dots, characteristic colour vanishing	3
	Yellow and mucous	4
Smell	Fresh, seaweedy, metallic	0
	Neutral	1
	Fishy, trace of thawing odour	2
	Obvious thawing odour, sour, trace of ammonia	3
	Strong ammonia, off‐odour	4
Gaping	No visible gaps	0
	Gaping less than 20% (1–3) longitudinal cracks	1
	Minor gaping on one area (20%) or >3 longitudinal cracks	2
	Some gaping, 25–75% of the fillet	3
	Deep cracks or gaping in more than 75% of the fillet	4
	Grade (0–14) TOTAL SCORE	

### Microbiological analysis

Microbiological parameters included analyses of total viable counts on iron agar (TVC‐IA), hydrogen sulfide (H_2_S)‐producing bacteria, coliforms, thermo‐tolerant coliforms and presumptive *Escherichia coli*, and *Listeria monocytogenes*. The microbiological analyses of the cod fillets were performed directly after filleting and throughout the cold storage period, and of the thawing water before and after thawing. The TVC‐IA of fillets was examined by aerobic cultivation on iron agar Lyngby (Oxoid), which also gives the number of H_2_S‐producing bacteria as black colonies, due to precipitation of iron sulfide (FeS).^26^, ^27^ Sample preparation was done according to the Nordic Committee on Food Analysis (NMKL) method;^28^ however, surface plating and incubation was carried out at 17 °C for 4–5 days. Black colonies and all colonies were enumerated and the results reported separately as the logarithm of colony‐forming units (CFU) per gram. For analyses of coliforms and presumptive *E. coli*, 20 g muscle tissue and 25 mL thawing media respectively was set up applying a 3 × 3‐tube most probable number (MPN) method.^29^, ^30^ The cod fillets were measured in triplicate (three individual fillets) and the thawing medium was measured in duplicate (two samples), and the results were reported as MPN per gram and MPN per millilitre, respectively. For the detection of *L. monocytogenes*, 25 g of muscle tissue was homogenised with 225 mL LF listeria dilution broth, according to the NMKL method,^31^ followed by analyses using miniVIDAS (BioMérieux), performed according to the protocol of the supplier.

### Proximate composition

The water, protein and lipid content of the fillets was determined directly after filleting. Moreover, the water content was monitored throughout the whole storage period. Water content was determined by difference in weight of homogenised muscle samples and thawing water before and after drying for 4 h at 103 ± 1 °C.^32^ Results were calculated as grams of water per 100 grams of sample. The total protein content of the fish muscle was estimated with the Kjeldahl method with the aid of a Digestion System 40, 1026 distillation unit (Tecator AB, Hoeganaes, Sweden) and calculated using total nitrogen (N) × 6.25. Total lipids were extracted from 25 g fish muscle (80 ± 1% water) with methanol/chloroform/0.88% KCl_(aq)_ (at 1/1/0.5, v/v/v) according to the method of Bligh and Dyer.^33^ The lipid content was determined gravimetrically and the results were expressed as grams of lipids per 100 grams of wet muscle.

### Total volatile bases nitrogen

The total volatile bases nitrogen (TVB‐N) was quantified by steam distillation (Struer TVN distillatory, Struers, Copenhagen) and titration, after extracting the fish muscle with 7.5% aqueous trichloroacetic acid solution.^34^ The distilled TVB‐N was collected in boric acid solution and then titrated with sulfuric acid solution. The results were expressed as milligrams of TVB‐N per 100 grams of muscle.

### Statistical analysis

Statistical analysis was performed using Microsoft Office Excel 2010 (Microsoft Inc., Redmond, WA, USA) and SigmaPlot 12.0 (Dundas Software Ltd., GmbH, Germany). One way analysis of variance, Duncan's test and Pearson's correlation were performed on the means of the values. The significant level was set at 95% (*P* < 0.05). Principal components analysis (PCA) was performed using Unscrambler^®^ (Version 10.2, CAMO ASA, Trondheim, Norway) to identify similarities and differences between samples. All variables were weighted with the inverse of the standard deviation to correct for different scales of the variables. Partial least square (PLS) regression models were developed to identify significant correlation between the quality index score method (sensory evaluation) and other chemical and microbial quality attributes. The quality index scores obtained were set as the *X*‐matrix, while each individual chemical or microbial quality parameter was set as the *Y*‐matrix. Full cross‐validation with uncertainty test was performed on all multivariate models.

## RESULTS AND DISCUSSION

### Temperature profiles

**Figure 3:**
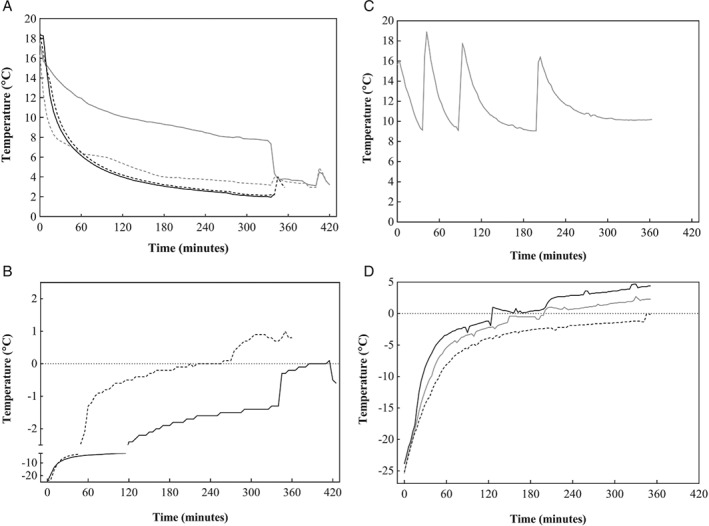
Temperature profiles. (A) The water with and without air circulation during thawing in fish tubs (grey: without air circulation; black: with air circulation; line: corner of the tub; dotted line: middle of the tub). (B) The fish muscles during the water thawing (dotted line: with air circulation; solid line: without air circulation). (C) The contact plates during thawing by using converted plate freezer. (D) The fish muscle during the contact thawing, showing the average of the core loggers (black line: <1 cm in the muscle; grey line: ∼1.5 cm in the muscle; dotted line: in the core).

The temperature profiles of the thawing media and the fish muscle during thawing in the water tubs are presented in Fig. 3. Water thawing with air circulation resulted in a more homogeneous thawing environment, as well as shorter thawing period, than water thawing without air circulation. The fish muscle thawed with air circulation reached a temperature of 0 °C after approximately 210 min, while the muscle thawed without air circulation reached the same temperature after approximately 380 min. The significant drop in temperature between 300 and 350 min can be explained by the blocks falling apart during thawing; as a result, individual fish were loose in the water. The surface of the blocks had a higher temperature than the core of the block, and when the blocks were separated the water was exposed to fish of much lower temperature, resulting in a rapid decrease of the water temperature. The time for the muscle to reach 0 °C when thawed in the converted plate freezer was approximately 230 min. When thawing seafood, an appropriate timescale is important to preserve quality and safety of the final product; and with perishable products like cod, thawing should be fast. The thawing time for all three methods in this study were in line with what could be expected from thawing in water or by air blast, and shorter than what could be expected from thawing in still air.^12^, ^35^

### Drip loss and cooking yield

The ability of the fish muscle to retain its natural water, and therefore its juiciness, is one of the quality criteria of fresh fish, especially from a consumer perspective. The drip loss during cold storage and cooking yield is presented in Table 3. Drip loss was based on the total weight of all fillets from each sampling point (1302–1778 g); therefore, only one measurement and no standard deviation is included. No obvious differences were observed between the different groups during 14 days of storage, and all measurements were below the 4–8% reported at the end of shelf life of fresh modified‐atmosphere packaged cod.^36^, ^37^ However, after 10 days of storage, the drip loss of the cod thawed in the converted plate freezer peaked at 2.8%, which was somewhat higher than the other experimental groups of water‐thawed cod. Drip loss has been linked to partial denaturation of proteins during processing, which cause lower WHC.^38^, ^39^ Accordingly, it can be assumed that the selected water thawing methods had slightly more preservative effects on the muscle quality than the contact plate‐thawing method.

**Table 3:** Fillet quality of frozen‐thawed Atlantic cod (G. morhua). Parameters were determined after thawing and after storage for up to 14 days. Comparisons of thawing in water with air circulation (air water), thawing in water without air circulation (still water) and plate thawing (plate)

	Day 0			Day 6			Day 10			Day 14		
Quality parameter	Air water	Still water	Plate	Air water	Still water	Plate	Air water	Still water	Plate	Air water	Still water	Plate
Water content (%)	81.7 ± 0.2	81.1 ± 0.2	81.3 ± 0.2	82.1 ± 0.5	81.9 ± 0.4	81.3 ± 0.3	81.5 ± 0.3	81.7 ± 0.4	80.8 ± 0.2	81.8 ± 0.2	81.8 ± 0.3	81.3 ± 0.7
Protein content (%)	16.9 ± 0.3	17.6 ± 0.3	17.3 ± 0.2	—	—	—	—	—	—	—	—	—
Lipid content (%)	01. ± 0.0	0.2 ± 0.2	0.1 ± 0.1	—	—	—	—	—	—	—	—	—
WHC (%)	77.7 ± 3.6	74.5 ± 2.8	80.6 ± 5.0	75.8 ± 1.6	77.7 ± 3.9	80.8 ± 0.9	78.7 ± 3.3	84.4 ± 1.8	90.0 ± 1.0	77.7 ± 7.0	89.0 ± 7.7	97.5 ± 2.3
Drip loss (%)	—	—	—	1.7	1.4	1.8	1.6	2.8	2.0	1.7	1.9	1.3
Cooking yield (%)	87.4 ± 3.8	85.9 ± 3.1	90.0 ± 1.4	81.6 ± 0.5	82.3 ± 5.2	85.9 ± 1.7	81.3 ± 2.9	78.4 ± 6.9	84.4 ± 1.8	90.7 ± 2.0	89.4 ± 3.5	92.2 ± 2.4
Texture (range: 0–2)	0.2 ± 0.4	0.3 ± 0.5	0.3 ± 0.5	0.3 ± 0.5	0.8 ± 0.4	0.3 ± 0.5	1.0 ± 0.0	0.7 ± 0.5	0.7 ± 0.5	1.2 ± 0.4	1.8 ± 0.4	1.5 ± 0.5
Colour (range: 0–4)	1.0 ± 0.0	0.8 ± 0.4	0.8 ± 0.4	1.4 ± 0.5	1.0 ± 0.0	1.3 ± 0.5	2.0 ± 0.0	1.5 ± 0.5	2.0 ± 0.0	2.8 ± 0.5	2.3 ± 0.5	2.0 ± 0.0
Smell (range: 0–4)	1.0 ± 0.0	1.0 ± 0.0	1.0 ± 0.0	1.3 ± 0.5	1.5 ± 0.5	1.3 ± 0.5	2.5 ± 0.5	2.7 ± 0.5	2.5 ± 0.5	3.0 ± 0.0	3.0 ± 0.0	3.7 ± 0.5
Gaping (range: 0–4)	0.6 ± 0.5	1.0 ± 0.6	1.0 ± 0.6	1.2 ± 0.4	0.7 ± 0.5	0.8 ± 0.4	1.8 ± 0.9	1.5 ± 0.5	1.2 ± 0.8	1.8 ± 0.8	1.8 ± 1.0	1.5 ± 0.5
TQI (range: 0–14)	2.8 ± 0.8	3.2 ± 0.8	3.2 ± 1.0	4.2 ± 1.2	4.0 ± 1.1	3.8 ± 0.8	7.5 ± 1.1	6.3 ± 1.2	6.3 ± 1.0	8.5 ± 1.2	9.0 ± 1.3	8.7 ± 1.2
TVC (log)	4.0 ± 0.3	4.8 ± 0.1	4.7 ± 0.3	6.4 ± 0.3	5.6 ± 0.1	6.9 ± 0.1	7.6 ± 0.4	8.0 ± 0.4	8.1 ± 0.2	8.3 ± 0.4	8.4 ± 0.0	8.4 ± 0.2
H_2_S (log)	2.4 ± 0.4	2.9 ± 0.4	2.8 ± 0.3	2.5 ± 2.2	3.8 ± 0.4	3.2 ± 2.9	6.2 ± 0.6	6.4 ± 0.1	6.2 ± 0.3	7.0 ± 0.3	7.0 ± 0.3	7.1 ± 0.3

H_2_S: H_2_S‐producing bacteria; TQI: total quality index.

The cooking yield is related to the amount of water lost by the muscle during cooking as a result of protein denaturation. After 6 and 10 days' storage, the cooking yield decreased slightly; however, no significant differences were observed between the different groups during 14 days of storage. The cooking yield was in line with previous findings of chilled stored fresh cod.^40^, ^41^

### Sensory evaluation

The quality index analysis scores of the cod fillets from different thawed H/G cod blocks, evaluated after 0, 6, 10, and 14 days' storage post‐thawing at 0–2 °C, are presented in Table 3. A high correlation was seen between the total quality index score and the storage time (r = − 0.95 ; P < 0.001), which shows that the attributes – namely, texture, smell, colour and gaping – gradually declined with extended storage time. However, no difference between experimental groups was observed each day of sampling. Of the individual quality attributes, only smell showed significant difference between experimental groups, where fillets from cod thawed in the converted plate freezer had a significantly higher score than with the fillets from the cod thawed in water. No difference was observed between the two different water‐thawing methods. The original scheme was developed for fresh cod fillets stored on ice (in plastic boxes without lids to allow air to access the fillets) and evaluated the skin of the fillets; however, for the current study, some of the attributes, such as skin appearance and blood, were estimated not to be relevant.

### Microbiological analysis

Water thawing is commonly used in the seafood industry and is used for a variety of products such as whole, H/G fish, fish blocks and shellfish.^12^ During thawing, fish and equipment are likely to be exposed to human contact; therefore, proper hygiene is crucial. In addition, the fish is in direct contact with the thawing medium, which can increase the risk of cross‐contamination. L. monocytogenes is a food‐borne pathogen occasionally present in the marine environment and may be found in raw and processed fish and seafood, or in the processing environment, and as such poses a potential biohazard to the consumer.^42^, ^43^, ^44^, ^45^ In this study, the presence of coliforms and thermo‐tolerant coliforms (presumptive E. coli) in the thawing media and in the cod fillets were examined. Additionally, the presence of L. monocytogenes in the thawing media and on the fillets was examined. No E. coli were detected in any of the fish samples, nor in the thawing media. In cod fillets thawed in the converted plate freezer, an average of 0.4 MPN g^1^ coliforms were detected. In the fillets thawed in the water bath without air circulation, an average of 1.2 MPN g^−1^ coliforms were detected. Coliforms detected in the thawing media with and without air circulation were 5.5 MPN mL^−1^ and 16.6 MPN mL^−1^ respectively. No L. monocytogenes was detected in any of the samples analysed. Coliform bacteria mainly originate from the intestines of warm‐blooded animals. Hence, assays for coliforms are often used as an indicator of the hygienic standards during food production.^46^ Based on a collection of current and former legislation and guidelines, Svanevik et al. 47 outlined and proposed an assessment scheme for fish, surfaces, and water samples for the pelagic fish industry, which could also be applied for the whitefish industry. These guidelines were set for quality, hygiene and safety by limits of heterotrophic plate counts, or total viable counts (TVC), faecal indicator organisms (thermo‐tolerant coliforms and enterococci) and L. monocytogenes respectively. The presence of coliforms in foods is undesirable; however, no limits were proposed for coliforms, because coliform counts are inadequate to differentiate between faecal and non‐faecal contamination. Considering the aforementioned assessment scheme, the hygiene conditions during the thawing process were considered good and there were no indications of impaired food safety.

**Figure 4:**
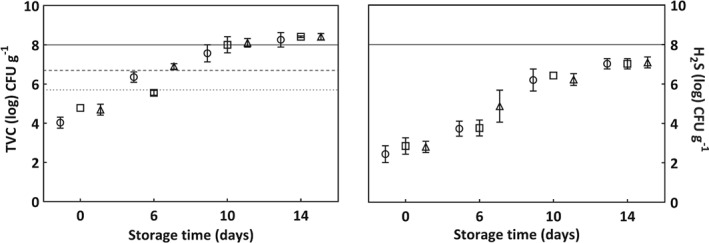
TVC‐IA and H_2_S‐IA (log CFU g^−1^) of cod fillets. Circles indicate thawing in water bath with air circulation, squares indicate thawing in water bath without air circulation, and triangles indicate thawing in converted plate freezer. Values are given as means of three samples with standard error of the mean as y‐error bars. The dotted line indicates good‐quality TVC‐IA limit (m = 5.7 log CFU g^−1^), the dashed line indicates acceptable‐quality TVC‐IA limit (M = 6.7 log CFU g^−1^)^37^ and the continuous line (8.0 log CFU g^−1^) indicates the level at which to consider a fish spoiled.^18^

TVC‐IA and H_2_S‐producing bacteria on iron agar (H_2_S‐IA) of cod fillets at day 0 after thawing, and after 6, 10, and 14 days of cold storage are shown in Fig. 4. As expected, both TVC‐IA and H_2_S‐IA increased with storage time (r = 0.96 and r = 0.92, respectively). High viable counts of fish samples are often related to an increased number of SSBs, causing off‐flavours associated with seafood spoilage, and possibly reducing the shelf life of the product.^19^ Microbial spoilage of fresh fish, stored aerobically in chilled conditions, is commonly seen if the number of SSBs exceeds 8.0 log CFU g^1^, ^17^ as indicated by the continuous line in Fig. 4. Considering counts of H_2_S‐producing bacteria, no samples were considered spoiled after 14 days of storage. In the aforementioned assessment scheme by Svanevik et al. 47 for evaluation of fresh fish, the two limits m and M were set, representing good (<m), acceptable (between m and M) and not acceptable (>M) conditions, as indicated by the dotted line (m = 5.7 log CFU g^−1^) and the dashed line (M = 6.7 log CFU g^−1^) in Fig. 4. Directly after thawing, at day 0, differences between the thawing methods were not significant. After 6 days of storage, the quality of the fish thawed in the water bath, with and without air circulation, was acceptable (TVC ‐ IA = 6.4 log CFU g^−1^) and good (TVC ‐ IA = 5.6 log CFU g^−1^) respectively. However, the quality of the fish thawed in the converted plate freezer was not acceptable, with TVC ‐ IA = 6.9 log CFU g^−1^.

Freezing the fish directly upon catch will not eliminate any microorganisms present, but will inhibit microbial and enzymatic spoilage, which again may extend the shelf life of the product. A study by Bonilla *et al*.^24^ estimated shelf life of 7–10 days post‐filleting, based on counts of H_2_S‐producing bacteria and sensory evaluation. The short shelf life in that study, compared with previous studies, may be due to the storage time of the whole fish from catch until filleting, which was from 3 to 5 days. When fish is frozen soon after catch, followed by optimal thawing, such spoilage during transport from catch to the processing plant may be diminished.

### Proximate composition and water holding capacity

Fillet quality parameters are summarised in Table 3, and the water, protein and lipid content of the fillets were all in the expected range of principal components in whitefish muscle.^48^ The water content of the thawed cod fillets ranged from 80.8% to 82.1% (Table 3), with no significant difference between the experimental groups. These findings demonstrate that no water uptake occurred during the water thawing where the fish was in direct contact with the thawing medium.

The WHC was affected throughout the storage period for water thawing without air circulation and plate thawing. The WHC of still water and plate‐thawed samples increased with time, which was the opposite of what would be expected. However, in recent studies we have revealed similar results (unpublished results). Further, this might be explained by the fact that these groups had lost most of their loosely bound water at day 14, with mainly the intracellular water remaining. Alternatively, it could be linked to the higher amount of TVB‐N, which also includes formaldehyde, causing protein denaturation and cross‐linking and textural changes. Water thawing with air circulation provided a more stable final product.

### Total volatile bases nitrogen

**Figure 5:**
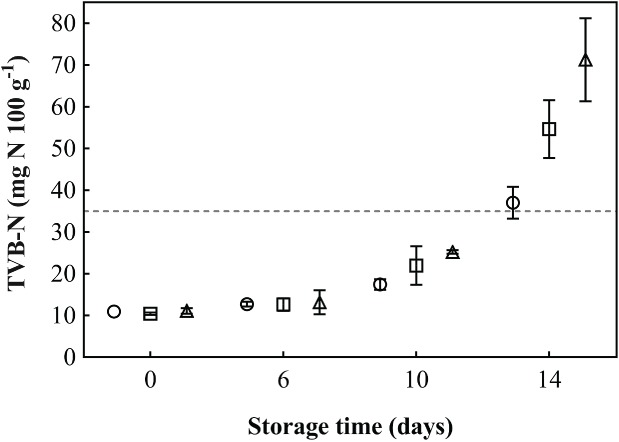
TVB‐N (mg N per 100 g wet muscle) of cod fillets from different thawed H/G cod blocks. Fillets were evaluated after 0, 6, 10 and 14 days' storage post‐thawing at 0–2 °C. The dashed line at TVB‐N level of 35 mg N 100 g^−1^ demonstrates the limits of acceptability.^19^, ^38^ Circles indicate thawing in water bath with air circulation, squares indicate thawing in water bath without air circulation, and triangles indicate thawing in converted plate freezer. Values are given as means of three samples with standard error of the mean as y‐error bars.

Changes in TVB‐N of cod fillets from different thawed H/G cod during cold storage are shown in Fig. 5. The TVB‐N increased more rapidly in fillets from fish thawed in the converted plate freezer, reaching a significantly higher level on day 14. This complies with the sensory evaluation, with a higher score (range [0–4] – the lower the better) for smell attribute of plate‐thawed cod. Based on the threshold limit of TVB‐N (35 mg N 100 g^−1^) for acceptability of consumption of gadoid species,^18^, ^49^ a shelf life of between 10 and 14 days was obtained for the experimental group thawed in water with air circulation, whereas for the other experimental groups a shelf life of at least 10 days was obtained. These results are in line with shelf‐life studies of fresh cod fillets stored under comparable conditions,^50^ suggesting that the fillets from thawed fish can be of similar quality to fresh fish. Shelf life of fresh cod fillets (processed from gutted, iced cod), stored at 0–1 °C, usually ranges between 10 and 13 days.^51^ Jónsson *et al*.^52^ and Sigurðsson^53^ compared shelf life of fresh and thawed cod fillets and showed that the quality of the frozen‐thawed fillets was comparable to fresh with regard to microbial analysis and TVB‐N. However, the WHC was better for fresh fillets than for frozen‐thawed fillets.

### Multivariate analysis

**Figure 6:**
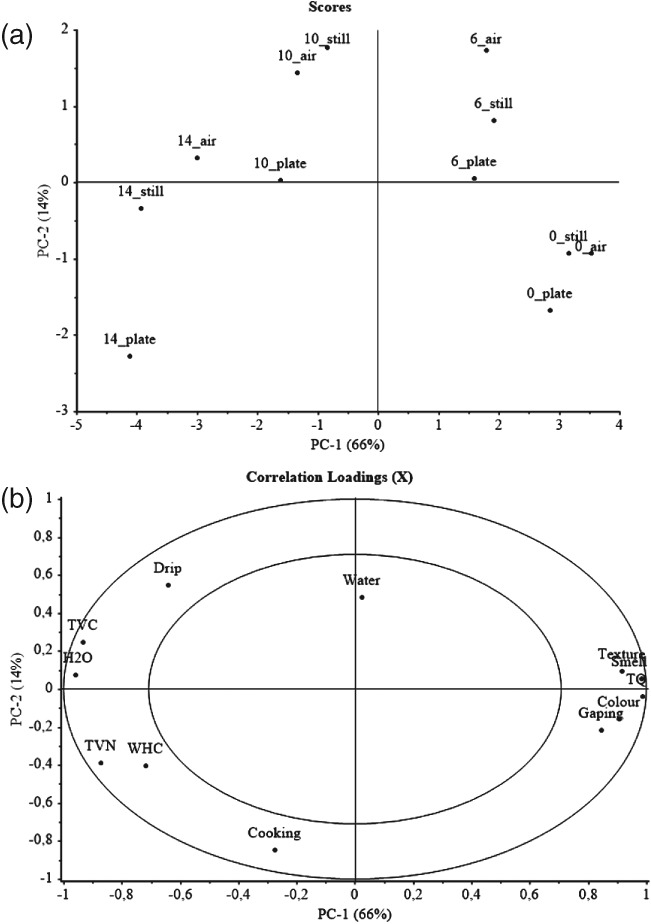
Scores (a) and correlation loadings (b) from PC‐1 and PC‐2 from weighted PCA of all fish samples analysed. All samples and analytical parameters were used. ‘Air’ indicates samples thawed in water with air circulation; ‘still’ indicates samples thawed in water without air circulation; and ‘plate’ indicates samples thawed in converted plate freezer. The number in the sample description indicates the storage time (days) at 0–2 °C.

Weighted PCA was carried out in order to gain an overview of the similarities and differences among the single variables (Fig. 6). All evaluated parameters were included in the analysis. The first two principal components described 80% of the variation between samples. The first principal component (PC‐1), representing 66% of the total variation, described primarily the effects of storage time, coupled to, for example, higher TVB‐N concentration and higher microbiological count at the end of the storage seen at the left‐hand end of PC‐1. The second principal component (PC‐2), accounting for 14% of the total variation, described the difference between different thawing methods. Accordingly, the difference between thawing methods was not significant until 14 days post‐filleting, where the water‐thawed samples with air circulation tended to retain the quality longer than other experimental groups.

Furthermore, the PCA and the Pearson correlation matrix (Table 4) suggested a strong negative relationship between the microbiological parameters (TVC and H_2_S‐producing bacteria) and the total quality index score (*r* = − 0.90 and *r* = − 0.95 respectively). These findings demonstrate the reliability of the sensory evaluation methods used in this study, indicating that the quality index score method has the potential to serve as a fresh fish quality parameter. In order to explore further the relationship between the quality index score method and the chemical and microbial quality parameters, PLS regression models were assessed to estimate from the sensory parameters (colour, texture, smell and gaping) the chemical and microbial quality of the samples. Performance of the PLS models for each quality parameter (TVC, H_2_S‐producing bacteria and TVB‐N) are listed in Table 5, including the coefficient of determination for cross validation $R_{v}^{2}$ and root‐mean‐square error of cross‐validation (RMSECV). In general, an accurate and robust model should have high $R_{v}^{2}$ and low RMSECV. However, it should be remarked that the performance of these models needs to be further validated with independent sample sets, to ensure the reliability and the accuracy of the models. For increased accuracy and robustness of the models, the number of samples analysed should ideally have been higher. However, several parameters were included in this trial, which may somewhat compensate for the low *n*. In future validation of the models, the number of samples analysed should be higher, for increased accuracy and robustness. Good regression models were developed to estimate the microbial quality of the cod fillets, while development of a model to estimate TVB‐N was not successful. These results demonstrate further the reliability of the quality index score method and its potential to serve as a fresh fish quality parameter.

**Table 4:** Correlation (Pearson) matrix for different parameters evaluated for cod fillets from different thawed H/G cod blocks. Fillets were evaluated after 0, 6, 10 and 14 days' storage post‐thawing at 0–2 °C

	H_2_S	TVB‐N	WHC	Water	Drip	Cooking	Texture	Colour	Smell	Gaping	TQI	ST
TVC	**0.90** a	**0.71**	**0.64**	−0.04	**0.83**	0.02	**−0.76**	**−0.87**	**−0.89**	**−0.80**	**−0.90**	**0.96**
H_2_S		**0.77**	**0.66**	−0.00	**0.63**	0.15	**−0.87**	**−0.87**	**−0.96**	**−0.84**	**−0.95**	**0.92**
TVB‐N			**0.82**	−0.02	0.32	0.57	**−0.88**	**−0.69**	**−0.89**	**−0.62**	**−0.84**	**0.79**
WHC				−0.38	0.43	0.35	−0.63	−0.44	**−0.74**	−0.36	**−0.62**	**0.62**
Water					−0.00	−0.20	−0.14	−0.09	0.04	−0.11	−0.05	0.13
Drip						−0.33	−0.44	**−0.65**	−0.58	−0.47	**−0.58**	**0.76**
Cooking							−0.35	−0.26	−0.29	−0.10	−0.27	0.12
Texture								**0.78**	**0.90**	**0.77**	**0.93**	**−0.87**
Colour									**0.87**	**0.84**	**0.93**	**−0.91**
Smell										**0.81**	**0.97**	**−0.94**
Gaping											**0.90**	**−0.81**
TQI												**−0.95**

H_2_S: H_2_S‐producing bacteria; TQI: total quality index score; ST: storage time.

a Bold figures denote statistical significance at *P* < 0.05.

**Table 5:** Performance of each PLS regression model developed

Estimated parameters	Factors	RMSEC	RMSECV	$R_{c}^{2}$	$R_{v}^{2}$
TVC	1	0.67	0.78	0.82	0.79
H_2_S‐producing bacteria	1	0.55	0.62	0.92	0.91
TVB‐N	2	6.90	12.1	0.87	0.65

RMSEC: root‐mean‐square error of calibration; RMSECV: root‐mean‐square error of cross‐validation; $R_{c}^{2}$: coefficient of determination in calibration; $R_{v}^{2}$: coefficient of determination in cross‐validation.

## CONCLUSION

To our best knowledge, this study presents one of the broadest comparative investigations of quality and safety factors after contact thawing, as well as water thawing with and without air circulation. These quality attributes include WHC, thawing loss, drip loss, cooking yield, sensory evaluation and microbiological analysis (including TVB‐N).

In this study, water thawing with air circulation provided the fastest thawing; however, in all, no significant differences were seen between the groups, and all three experimental groups retained the quality of the cod fillets. The hygiene conditions during the trial were considered good, and there were no indications of impaired food safety. Considering all parameters, including the evaluation of TVB‐N and microbial quality, a shelf life of the fillets during chilled storage between 10 and 14 days post‐filleting, is obtainable.

The results demonstrate that optimal thawing of fish frozen at sea may provide the industry with a stable supply of raw materials, without compromising quality and safety of the final product.

## References

[1] Adams MR and Moss MO , Microbiology of primary food commodities, in Food Microbiology. Royal Society of Chemistry, Cambridge, UK, pp. 119–125 (1995).

[2] Ashie INA , Smith JP and Simpson BK , Spoilage and shelf‐life extension of fresh fish and shellfish. Crit Rev Food Sci Nutr 36:87–121 (1996). 8747101 10.1080/10408399609527720

[3] Ghaly AE , Dave D , Budge S and Brooks M , Fish spoilage mechanisms and preservation techniques: review. Am J Appl Sci 7:859–877 (2010).

[4] Sampels S , The effects of storage and preservation technologies on the quality of fish products: a review. J Food Process Preserv 39:1206–1215 (2015).

[5] Li B and Sun D‐W , Novel methods for rapid freezing and thawing of foods – a review. J Food Eng 54:175–182 (2002).

[6] Shokr M and Sinha N , Sea Ice: Physics and Remote Sensing. John Wiley & Sons (2015).

[7] Baygar T , Alparslan Y and Çaklı Ş , Effects of multiple freezing and refrigerator thawing cycles on the quality changes of sea bass (*Dicentrarchus labrax*). Iran J Fish Sci 12:289–300 (2013). 10.1007/s13197-014-1373-z PMC4444873 26028727

[8] Ersoy B , Aksan E and Ozeren A , The effect of thawing methods on the quality of eels (*Anguilla anguilla*). Food Chem 111:377–380 (2008). 26047438 10.1016/j.foodchem.2008.03.081

[9] Genc IY , Esteves E , Anibal J and Diler A , Effects of different thawing methods on the quality of meagre fillets. Ankara Univ Vet Fak Derg 62:153–159 (2015).

[10] Mousakhani‐Ganjeh A , Hamdami N and Soltanizadeh N , Effect of high voltage electrostatic field thawing on the lipid oxidation of frozen tuna fish (*Thunnus albacares*). Innovative Food Sci Emerging Technol 36:42–47 (2016).

[11] Wang H , Luo Y , Shi C and Shen H , Effect of different thawing methods and multiple freeze–thaw cycles on the quality of common carp (*Cyprinus carpio*). J Aquat Food Prod Technol 24:153–162 (2015).

[12] Archer M , Edmonds M and George M , Seafood Thawing, Seafish Research & Development, SR598 (2008).

[13] Haugland A , Industrial thawing of fish: to improve quality, yield and capacity. PhD thesis, Department of Energy and Process Engineering, Norwegian University of Science and Technology Trondheim, Norway (2002).

[14] Backi CJ , Leth J and Gravdahl JT , Optimal boundary control of a contact thawing process for foodstuff. IFAC‐PapersOnLine 49:183–188 (2016).

[15] Pedrosa‐Menabrito A and Regenstein JM , Shelf‐life extension of fresh fish – a review part III – fish quality and methods of assessment. J Food Qual 13:209–223 (1990). 10.1111/j.1745-4557.1988.tb00872.x PMC7166583 32336837

[16] Backi CJ , Methods for (industrial) thawing of fish blocks: a review. J Food Proc Eng 2017: e12598 (2017).

[17] Gram L and Huss HH , Microbiological spoilage of fish and fish products. Int J Food Microbiol 33:121–137 (1996). 8913813 10.1016/0168-1605(96)01134-8

[18] Ólafsdóttir G , Lauzon HL , Martinsdóttir E , Oehlenschláuger J and Kristbergsson K , Evaluation of shelf life of superchilled cod (*Gadus morhua*) fillets and the influence of temperature fluctuations during storage on microbial and chemical quality indicators. J Food Sci 71:S97–S109 (2006).

[19] Gram L and Dalgaard P , Fish spoilage bacteria – problems and solutions. Curr Opin Biotechnol 13:262–266 (2002). 12180103 10.1016/s0958-1669(02)00309-9

[20] Stroud GD , Rigor in fish: the effect on quality Torry Advisory Note No. 36, Ministry of Technology, Torry Research Station, Aberdeen (1969).

[21] Backi CJ , Modeling, estimation and control of freezing and thawing processes: theory and applications. PhD thesis, Department of Engineering Cybernetics, Norwegian University of Science and Technology (NTNU), Trondheim, Norway (2015).

[22] Backi CJ , Bendtsen JD , Leth J and Gravdahl JT , A heat equation for freezing processes with phase change: stability analysis and applications. Int J Control 89:833–849 (2016).

[23] Eide O , Børresen T and Strøm T , Minced fish production from capelin (*Mallotus villosus*). A new method for gutting, skinning and removal of fat from small fatty fish species. J Food Sci 47:347–349 (1982).

[24] Bonilla AC , Sveinsdottir K and Martinsdottir E , Development of quality index method (QIM) scheme for fresh cod (*Gadus morhua*) fillets and application in shelf life study. Food Control 18:352–358 (2007).

[25] ISO 8586‐1:1993, Sensory analysis – General guidance for the selection, training and monitoring of assessors. Part 1: selected assessors. The International Organization for Standardization (ISO), Geneva (1993).

[26] Gram L , Evaluation of the bacteriological quality of seafood. Int J Food Microbiol 16:25–39 (1992). 1389992 10.1016/0168-1605(92)90123-k

[27] Gram L , Trolle G and Huss HH , Detection of specific spoilage bacteria from fish stored at low (0°C) and high (20°C) temperatures. Int J Food Microbiol 4:65–72 (1987).

[28] NMKL 184 , 2006, Aerobic count and specific spoilage organisms in fish and fish products. Nordic Committee on Food Analysis (NMKL), Oslo (2006).

[29] Kornacki JL and Johnson JL , Enterobacteriaceae, coliforms, and *Escherichia coli* as quality and safety indicators, in Compendium of Methods for the Microbiological Examination of Foods, 4th edition, ed. Downes FP and Ito K American Public Health Association, pp. 69–82 (2001).

[30] NMKL 96 , 4 edition, Coliform bacteria, thermotolerant coliform bacteria and *E. coli*, two MPN methods for fresh and frozen seafood. Nordic Committee on Food Analysis (NMKL), Oslo (2009).

[31] NMKL 136 , 5th edition , 2010, *Listeria monocytogenes*. Detection in foods and feeding stuffs and enumeration in foods. Nordic Committee on Food Analysis (NMKL), Oslo, Norway (2010).

[32] ISO 6496:1999, Animal feeding stuffs – Determination of moisture and other volatile matter content. International Organization for Standardization (ISO), Geneva (1999).

[33] Bligh E and Dyer W , A rapid method of total extraction and purification of lipids. Can J Biochem Physiol 37:911–917 (1959). 13671378 10.1139/o59-099

[34] Malle P and Tao SH , Rapid quantitative determination of trimethylamine using steam distillation. J Food Prot 50:756–760 (1987). 10.4315/0362-028X-50.9.756 30978808

[35] Martinsdóttir E and Magnússon H , Keeping quality of sea‐frozen thawed cod fillets on ice. J Food Sci 66:1402–1408 (2001).

[36] Dalgaard P , Gram L and Huss HH , Spoilage and shelf‐life of cod fillets packed in vacuum or modified atmospheres. Int J Food Microbiol 19:283–294 (1993). 8257657 10.1016/0168-1605(93)90020-h

[37] Guldager HS , Boknaes N , Osterberg C , Nielsen J and Dalgaard P , Thawed cod fillets spoil less rapidly than unfrozen fillets when stored under modified atmosphere at 2 degrees C. J Food Prot 61:1129–1136 (1998). 9766063 10.4315/0362-028x-61.9.1129

[38] Mackie I , The effects of freezing on flesh proteins. Food Reviews International 9:575–610 (1993).

[39] Shenouda SY , Theories of protein denaturation during frozen storage of fish flesh, in Advances in Food Research, vol. 26, ed. by Chichester CO, EM Mrak and GF Stewart. Academic Press, New York, pp. 275–308 (1980).

[40] Gao HY , Methods of pre‐cooling for fresh cod (*Gadus morhua*) and influences on quality during chilled storage at −1.5 °C. Final report, The United Nations University (UNU), Fisheries Training Programme, Reykjavik, Iceland (2007).

[41] Karlsdottir MG , Application of additives in chilled and frozen whitefish fillets: effects on chemical and physicochemical properties. MSc thesis, School of Health Sciences, University of Iceland, Reykjavik, Iceland, (2009).

[42] Embarek PKB , Presence, detection and growth of *Listeria monocytogenes* in seafoods: a review. Int J Food Microbiol 23:17–34 (1994). 7811570 10.1016/0168-1605(94)90219-4

[43] Huss HH , Jørgensen LV and Vogel BF , Control options for *Listeria monocytogenes* in seafoods. Int J Food Microbiol 62:267–274 (2000). 11156271 10.1016/s0168-1605(00)00347-0

[44] Jami M , Ghanbari M , Zunabovic M , Domig KJ and Kneifel W , *Listeria monocytogenes* in aquatic food products – a review. Compr Rev Food Sci Fod Saf 13:798–813 (2014).

[45] Jørgensen LV and Huss HH , Prevalence and growth of *Listeria monocytogenes* in naturally contaminated seafood. Int J Food Microbiol 42:127–131 (1998). 9706805 10.1016/s0168-1605(98)00071-3

[46] Noble RT , Lee IM and Schiff KC , Inactivation of indicator micro‐organisms from various sources of faecal contamination in seawater and freshwater. J Appl Microbiol 96:464–472 (2004). 14962126 10.1111/j.1365-2672.2004.02155.x

[47] Svanevik CS , Roiha IS , Levsen A and Lunestad BT , Microbiological assessment along the fish production chain of the Norwegian pelagic fisheries sector – results from a spot sampling programme. Food Microbiol 51:144–153 (2015). 26187839 10.1016/j.fm.2015.05.016

[48] Murray J and Burt JR , The composition of fish. Torry Advisory Note No. 38, Ministry of Technology, Torry Research Station, Aberdeen (1969).

[49] 95/149/EC CD , Commission Decision of 8 March, 1995 fixing the total volatile basic nitrogen (TVB‐N) limit values for certain categories of fishery products and specifying the analysis methods to be used. Off J Eur Communities L 097:0084–0087 (1995).

[50] Lauzon HL , Magnússon H , Sveinsdóttir K , Gudjónsdóttir M and Martinsdóttir E , Effect of brining, modified atmosphere packaging, and superchilling on the shelf life of cod (*Gadus morhua*) loins. J Food Sci 74:M258–M267 (2009). 19723210 10.1111/j.1750-3841.2009.01200.x

[51] Lauzon HL , Margeirsson B , Sveinsdóttir K , Guðjónsdóttir M , Karlsdóttir MG and Martinsdóttir E , Overview on fish quality research. Impact of fish handling, processing, storage and logistics on fish quality deterioration Matís report 39‐10, Matís, Reykjavik, Iceland (2010).

[52] Jónsson Á , Karlsdóttir MG , Sigurðsson E and Arason S , Þíðing á sjófrystum flökum [Thawing of frozen cod fillets]. Matís report 14‐16, Matís, Reykjavik, Iceland (2016).

[53] Sigurðsson E , Effects of nematodes on processing of cod. MSc thesis, University of Iceland, Reykjavik, Iceland (2016).

